# Burr Hole Surgery for Drainage of Chronic and Subacute Subdural Hematomas: Low Recurrence Rate in a Single Surgeon Cohort

**DOI:** 10.7759/cureus.19288

**Published:** 2021-11-05

**Authors:** Orlando De Jesus, Andres E Monserrate

**Affiliations:** 1 Neurosurgery, University of Puerto Rico, Medical Sciences Campus, San Juan, PRI

**Keywords:** burr hole, chronic subdural hematoma, neurosurgery, recurrence, subacute subdural hematoma, subdural hematoma

## Abstract

Background: A complication after surgical intervention for chronic and subacute subdural hematoma drainage is the recurrence of hematoma, often requiring repeat surgical management. Recurrence rates varied widely across the published series, which may partially be due to different technical strategies used by the surgeons involved in the study. We decided to review our patients with chronic and subacute subdural hematomas that were surgically managed with a burr hole procedure by a single surgeon to compare recurrence rates with the evidence available.

Methods: A retrospective review of the medical records was performed on the patients who underwent burr hole surgical intervention to drain a chronic or subacute subdural hematoma between April 1995 and March 2020. All patients were under the care of a single surgeon at an academic institution. Variables analyzed included age, sex, laterality of the hematoma, and recurrence.

Results: During the selected timeframe, 610 cases were identified. There were 35 cases of recurrence of the hematoma. The recurrence rate after burr hole drainage was 5.73%.

Conclusion: Surgical drainage of chronic and subacute subdural hematoma via burr hole using consistent stepwise management is associated with a relatively low recurrence rate in our single surgeon patient cohort.

## Introduction

Chronic subdural hematoma (CSDH) and subacute subdural hematoma (SASDH) are among the most common diagnoses for neurosurgical consultation at our emergency department. In symptomatic patients, both hematomas are primarily managed surgically via burr hole drainage and subdural irrigation. A common complication after surgical intervention for chronic/subacute subdural hematoma (C/SA-SDH) drainage is hematoma recurrence, often requiring repeat surgical management, more extended hospitalization, and sometimes, increased morbidity and mortality.

A literature review showed that recurrence rates for C/SA-SDH ranged between 0% and 20% [1-10]. Recurrence rates varied widely, which may partially be due to different technical strategies used by the surgeons involved. The purpose of this study was to describe an adult population of patients who underwent burr hole surgery for drainage of C/SA-SDHs between 1995 and 2020 by a single neurosurgeon and compare the recurrence rates with the available literature.

## Materials and methods

The operative experience with C/SA-SDH operated during 25 years by the senior author (OD) at the University of Puerto Rico Medical Center, a large academic tertiary medical center, was reviewed. A retrospective study was conducted using C/SA-SDH cases obtained from the medical record database between 1995 and 2020. Data of 610 patients treated for C/SA-SDHs by a single surgeon during the selected timeframe were obtained. The parameters analyzed were sex, age, laterality of the hematoma, and recurrence. We defined a recurrence as the reappearance of the subdural collection causing mass effect and new clinical symptoms within a three-month follow-up period. The overall recurrence rate was obtained and compared with those reported in the literature. We divided the patients into three equal periods during the selected time frame to study the recurrence rate among those periods. This was done to study the recurrence rate of the senior surgeon during the beginning, middle, and end of his career. This study received institutional review board approval (#B0940221).

## Results

**Figure 1:**
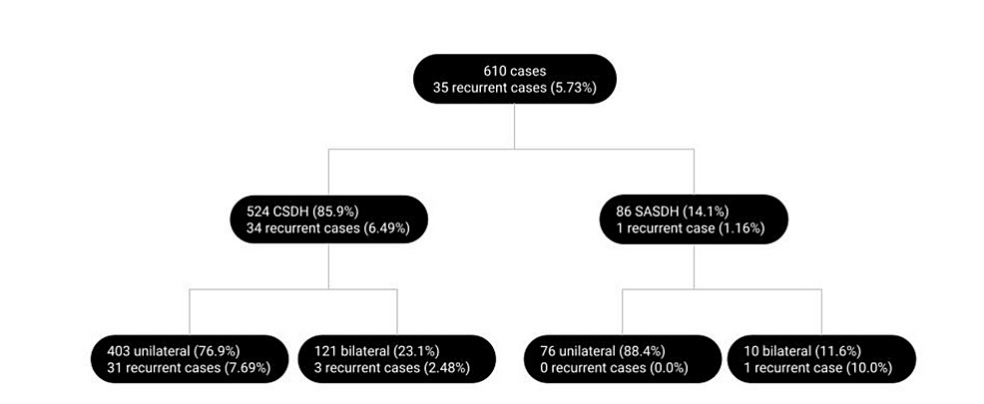
Flow diagram of chronic subdural hematoma and subacute subdural hematoma cases. CSDH: chronic subdural hematoma; SASDH: subacute subdural hematoma

We identified 610 cases from the medical records database for the selected timeframe (Figure 1). We identified 524 CSDH cases, which were unilateral in 403 patients and bilateral in 121 (23.1%). There were 86 SASDH cases, which were unilateral in 76 patients and bilateral in 10 (11.6%). In total, bilateral cases occurred in 131 patients (21.5%). There were 35 cases of recurrence of the hematoma. The overall recurrence rate for C/SA-SDHs following burr hole drainage was 5.73% (35/610). One recurrent case was after a SASDH (1.16%), and 34 were after a CSDH (6.49%). Twelve patients with recurrent hematoma were female (34.3%), and 23 were male (65.7%). The age of the patients with recurrent hematoma ranged from 43 to 88 years, with a median age of 74 years. Fourteen patients had a recurrent hematoma on the right side (40%) and 17 were on the left side (48.6%). Four patients had bilateral recurrence (11.4%), the initial collection was bilateral in all of them. When the selected time frame of 25 years was divided into three equal periods, the recurrence rate of the senior surgeon during the beginning of his career was 6.6%, at the middle was 6.3%, and at the end was 4.8%.

All patients were treated with a single or double burr hole performed over the collection. During the 25 years, the technique was not changed, and the same standardized stepwise method was used. The patient’s head was positioned so that the burr hole was at the highest possible point on the cranium to fill the cavity with saline and reduce the amount of pneumocephalus. Bone wax was applied to the bone borders to prevent postoperative blood oozing. Careful dural edge coagulation was performed to avoid epidural bleeding. If the hematoma was multilayer, each layer was individually opened and irrigated. Spontaneous hematoma drainage was initially allowed and then we began irrigation with a warm sterile saline solution into four quadrants using an 8F feeding tube and disposable 60 mL syringes starting at the posterior quadrant and moving clockwise until each quadrant irrigation wash became clear. Irrigation was again done at the posterior quadrant to remove any residual debris. The cavity was filled with warm sterile saline solution. The feeding tube was used as a subdural drainage tube, which was pulled through a separate skin incision. The feeding tube was connected to a sterile collecting bag without suction. The tip of the feeding tube drainage inside the cavity was placed at the area with the largest space, which was anterior in most of the cases. The anterior position of the subdural drain helped to eliminate the air collection as the brain re-expanded. The burr hole was not covered with any foam material to allow subgaleal fluid absorption. The wound was closed using a two-layer closure. The subdural drain was kept for at least 24 hours but no more than 48 hours. A head computed tomographic (CT) scan was performed on the first postoperative day to ensure adequate hematoma drainage. We found that this postoperative CT scan was unnecessary unless the patient showed any new neurological deficit; however, we routinely performed it for academic teaching purposes. A head CT scan was performed in all patients at the end of the three-month follow-up period.

## Discussion

In this study, we presented the C/SA-SDH cases managed by one neurosurgeon during a 25-year period. Based on our results, we believe that the surgical technique and experience of the surgeon are directly correlated with recurrence rates of C/SA-SDHs. Although not technically complicated, successful drainage of C/SA-SDH requires finesse and composure. It is crucial to develop a standardized stepwise method for surgical intervention to ensure the best outcomes for the patients. Insufficient initial hematoma drainage or inadequate hemostasis may lead to adverse outcomes.

Our overall recurrence rate of C/SA-SDH following burr hole drainage was 5.73%. This recurrence rate was in the lower range compared to rates reported in the literature. A study of only 47 patients with unilateral CSDHs operated using two enlarged (2 cm) burr holes was reported by Abdelfatah, showing no recurrence [1]. A systematic review of CSDHs showed an overall recurrence rate of 11.7% (4-31%) after burr hole drainage [11]. In a study comparing the use of atorvastatin with a group in which it was not used for the management of CSDH, Tang et al. reported a 4.8% recurrence rate when atorvastatin was used; however, the overall recurrence rate of the study was 9% [12]. Yadav et al. reported a recurrence rate of 3.57% in patients with subgaleal suction drainage compared to those without drainage; however, the overall recurrence rate of the study was 6.5% [13]. In a small series of CSDHs, Kwon et al. noted that the drained volume was a significant factor for recurrency, with no recurrence if the drained volumes were more than 200 m [14]. They showed that mixed-density hematomas had higher rates of recurrence with an overall 4.0% recurrence rate. However, Shen et al. showed that the hematoma density was not a significant factor for recurrence [15]. Unilateral hematomas have been shown to have a higher recurrence rate than bilateral hematomas [14,16]. Yu et al. showed that prolonged drainage for three or more days had a significantly lower recurrence rate with no increase in the frequency of infection [17]. We did not use drainage for more than 48 hours, and most of our patients were discharged on the second postoperative day or transferred to a rehabilitation facility.

In 1998, Suzuki et al. reported a recurrence rate of 3.2%, in which the majority of the patients were treated with a close drainage system without irrigation of the cavity [2]. Their series has the lowest reported recurrence rate in the literature for studies with at least 100 patients. The recurrence rate in the series by Kotwica et al. is the second-lowest reported in the literature (3.7%) [3]. In our patient cohort, surgical drainage of C/SA-SDH via burr hole was associated with a low recurrence rate (5.7%). This rate is the third-lowest recurrence rate among all published series with at least 100 patients. Some surgeons have attributed a higher recurrence to the type of procedure used. In the series by Raghavan et al., a burr hole had a greater recurrence rate (15.5%) than a craniotomy (7.5%) [18]. However, Shim et al. found a higher incidence with craniotomy (47%) than burr hole (13%) [9]. Haron et al. found no significant difference associated with the type of procedure used [7]. In our cohort, no patient required a craniotomy to drain a C/SA-SDH at the initial operation or to manage a recurrence. In a Canadian national survey to explore the management of C/SA-SDHs, surgeons preferred one burr-hole (35.5%) or two burr-holes (49.5%) to craniotomy (4.7%) or twist-drill (9.3%) as the procedure of choice for initial treatment [19]. However, for recurrent subdural hematomas, craniotomy (43.3%) and two burr-holes (35.1%) were preferred. In their study, most surgeons agreed on irrigating the subdural cavity.

We request a follow-up head CT scan at the three-month postoperative time unless the patient develops recurrence symptoms before. In the study by Lutz et al., all recurrences developed within three months [20]. This three-month time frame could be used as the estimated duration for the clinical follow-up period unless the patient had a reoperation, requiring a new three-month follow-up. Shen et al. showed that anticoagulation, brain atrophy, old age, a large amount of pneumocephalus, drainage volume >100 mm, and midline shift >11 mm were important factors for recurrence [21]. In our study, old age was a factor for recurrence. Some authors have shown no increased recurrence risk with preoperative antiplatelet or anticoagulation use [5,22]. The use of postoperative drains has been shown to reduce recurrence rates significantly [23]. We used a drain in all of our cases unless there was a complete reexpansion of the brain. Licci et al. showed no difference in recurrence using postoperative deep vein thrombosis prophylaxis given before 48 hours [6]. We started postoperative deep vein thrombosis prophylaxis 48 hours following the procedure after a head CT scan confirmed adequate drainage of the hematoma. We think that consistent management of C/SA-SDH can produce a low recurrence rate. Using a combination of operative steps, Abdelfatah produced excellent postoperative results [1]. Our study included patients from a single center operated by a single surgeon providing consistent management for patients. In our study, the senior surgeon was present in all operations, providing consistent treatment and supervision in an academic setting.

This study has some limitations. Despite no patient selection bias, the surgeon used a burr hole procedure on all cases of C/SA-SDH; however, this type of management may not be applied to all institutions as some surgeons may prefer other types of procedures. For this study, we did not analyze other factors that may influence the recurrence rate.

## Conclusions

Consistent management of C/SA-SDH can produce excellent results with very low recurrence rates. Surgical drainage of C/SA-SDH via burr hole is associated with a relatively low recurrence rate in our single surgeon patient cohort. Recurrence of the hematoma is more common in patients with older age. SASDHs have a significantly lower recurrence rate compared to CSDHs.
